# Supplementary figures and images for: Construction and validation of co-expression vector for rice alpha tubulin and microtubule associated protein respectively fused with fluorescent proteins
Source: PeerJ. 2024 Sep 26;12:e18118. doi: 10.7717/peerj.18118 (PMC11439384; doi:10.7717/peerj.18118)

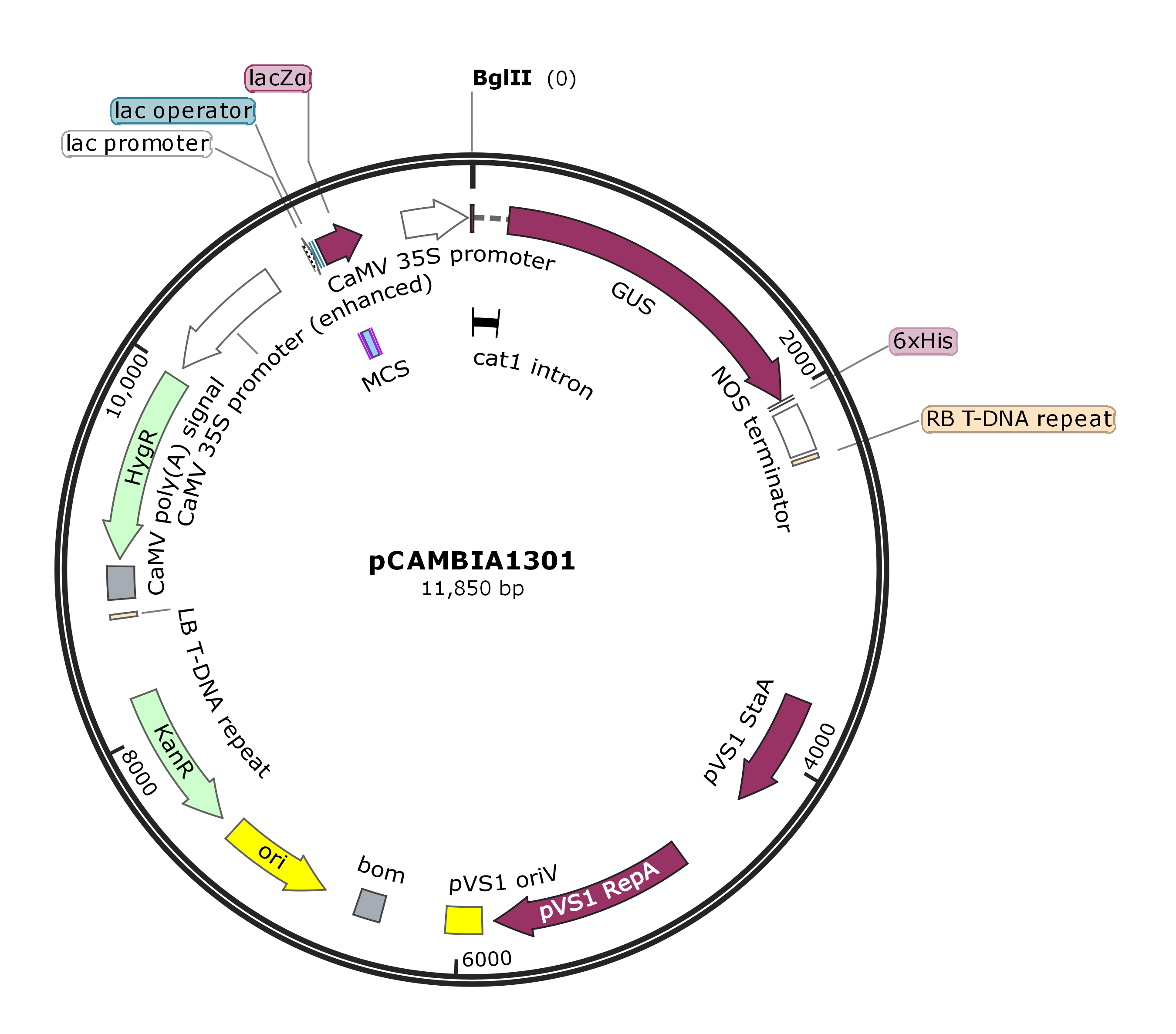

Supplement: Supplemental Information 1 [file peerj-12-18118-s001.zip › The original images for Fig.1/Fig.1a.png]

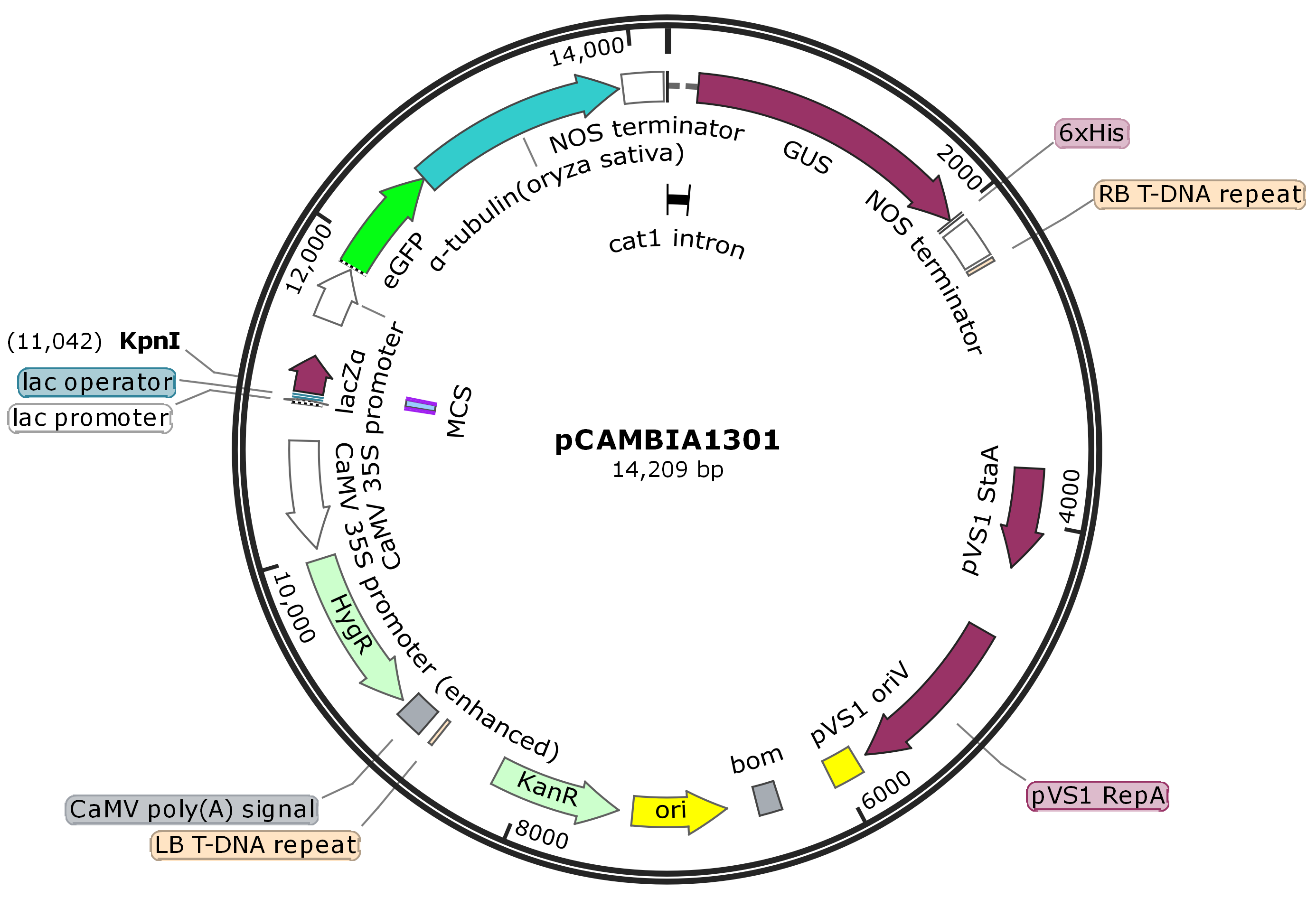

Supplement: Supplemental Information 1 [file peerj-12-18118-s001.zip › The original images for Fig.1/Fig.1b.png]

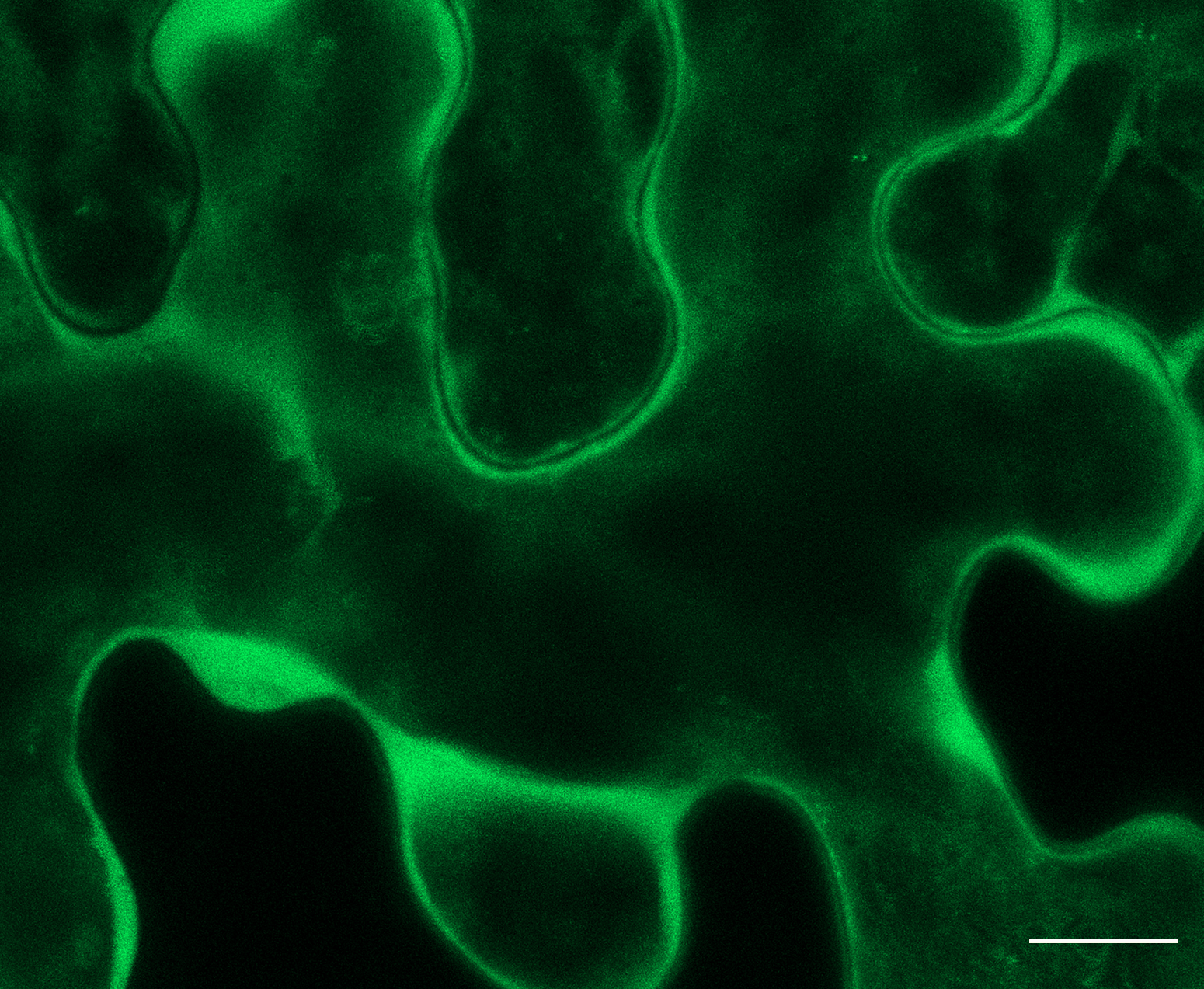

Supplement: Supplemental Information 1 [file peerj-12-18118-s001.zip › The original images for Fig.1/Fig.1c.tif]

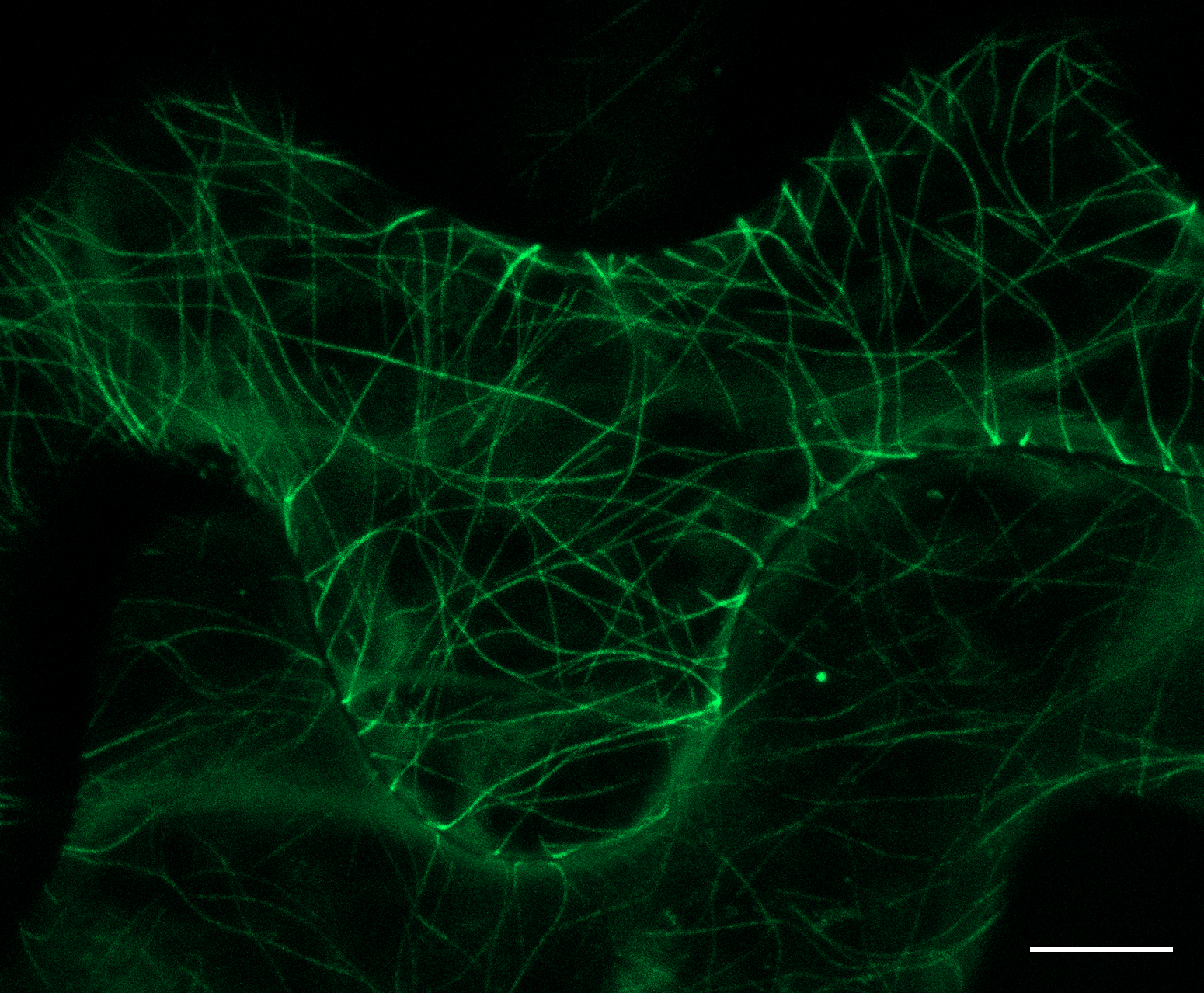

Supplement: Supplemental Information 1 [file peerj-12-18118-s001.zip › The original images for Fig.1/Fig.1d.tif]

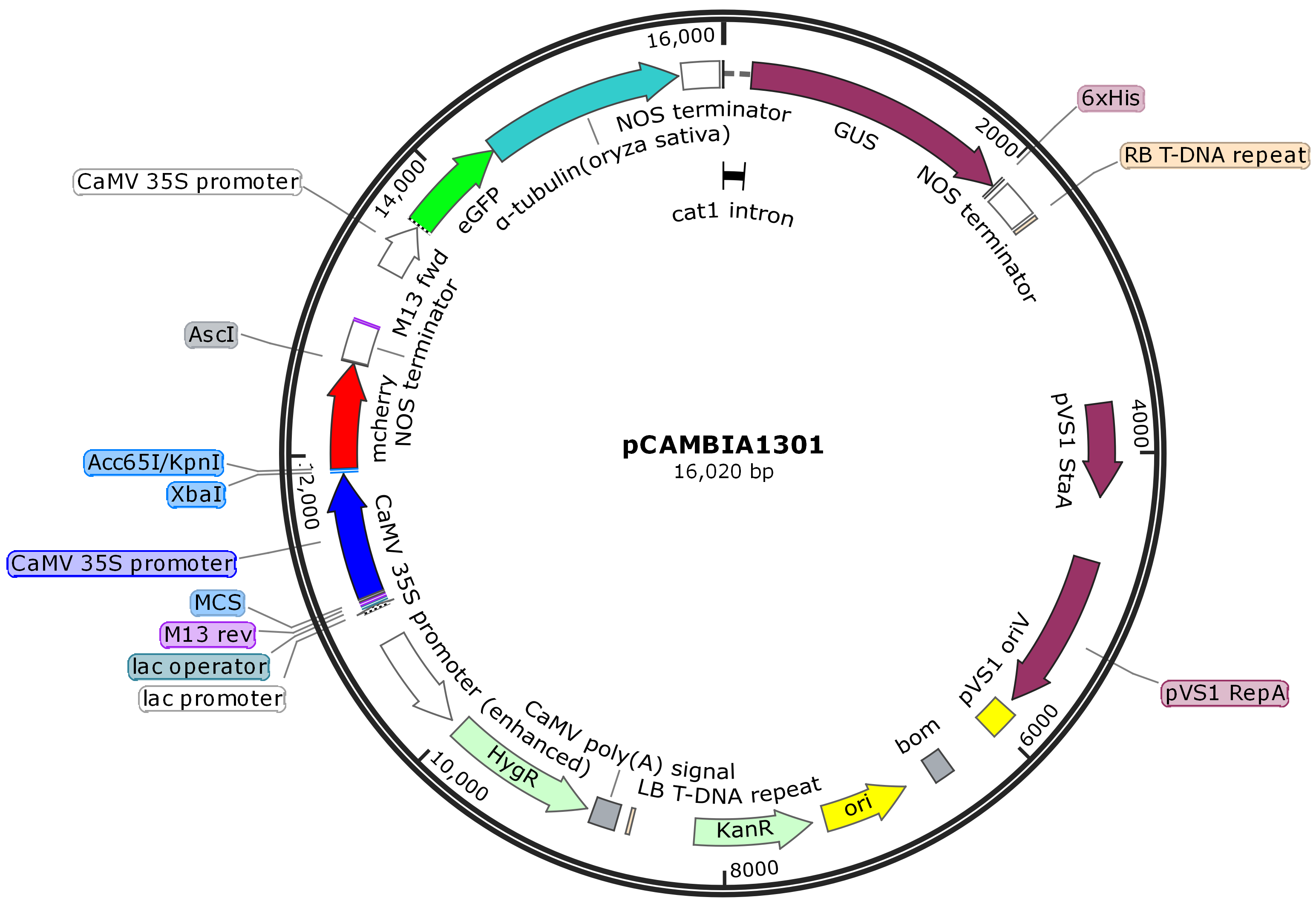

Supplement: Supplemental Information 2 [file peerj-12-18118-s002.zip › The original images for Fig.2/Fig.2a.png]

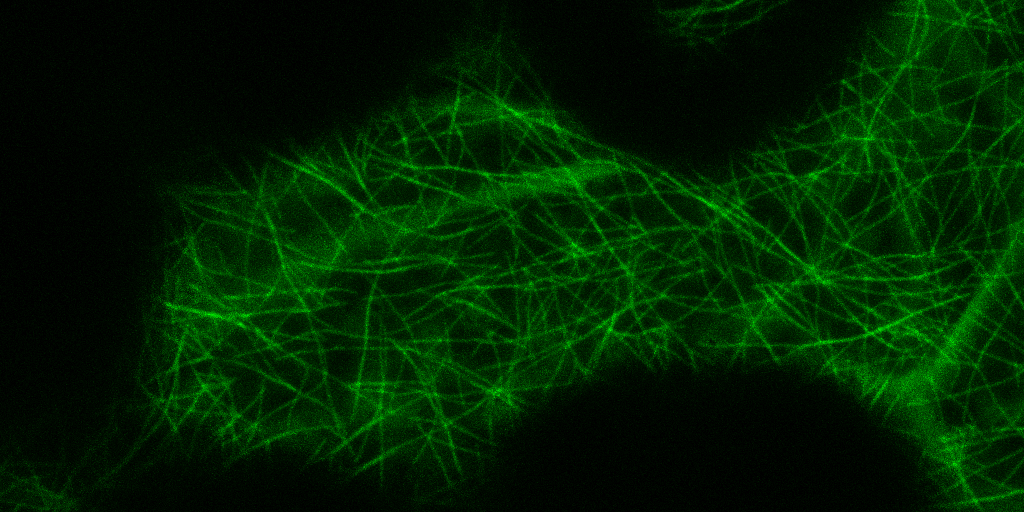

Supplement: Supplemental Information 2 [file peerj-12-18118-s002.zip › The original images for Fig.2/Fig.2b.tif]

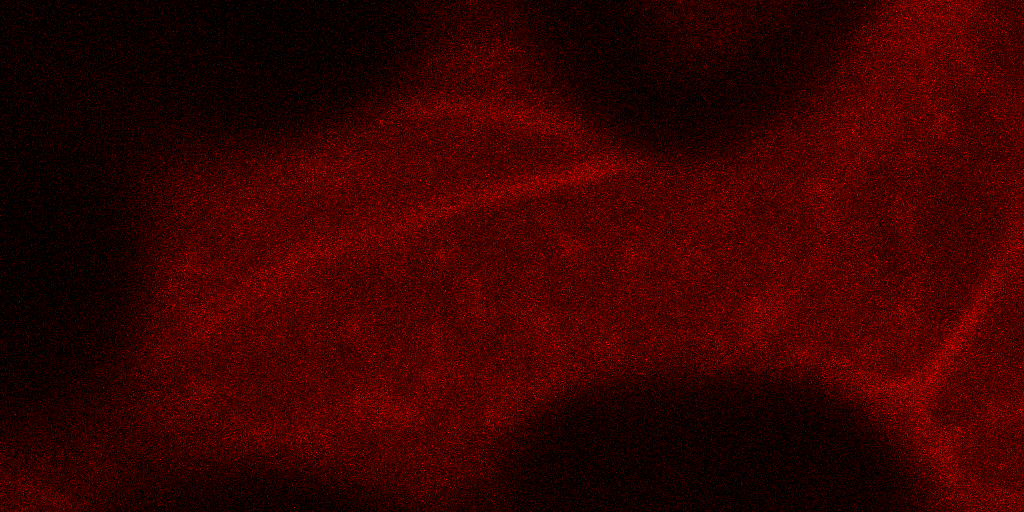

Supplement: Supplemental Information 2 [file peerj-12-18118-s002.zip › The original images for Fig.2/Fig.2c.tif]

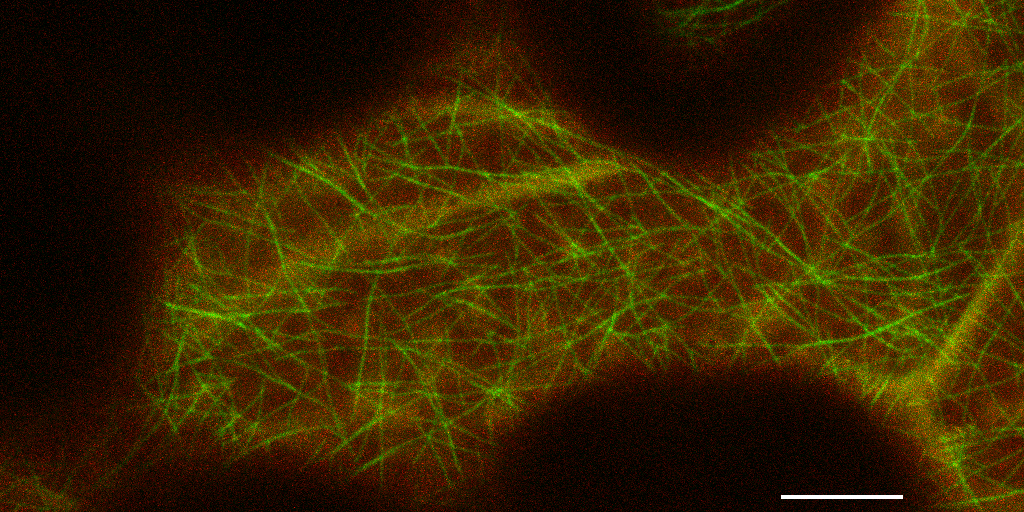

Supplement: Supplemental Information 2 [file peerj-12-18118-s002.zip › The original images for Fig.2/Fig.2d.tif]

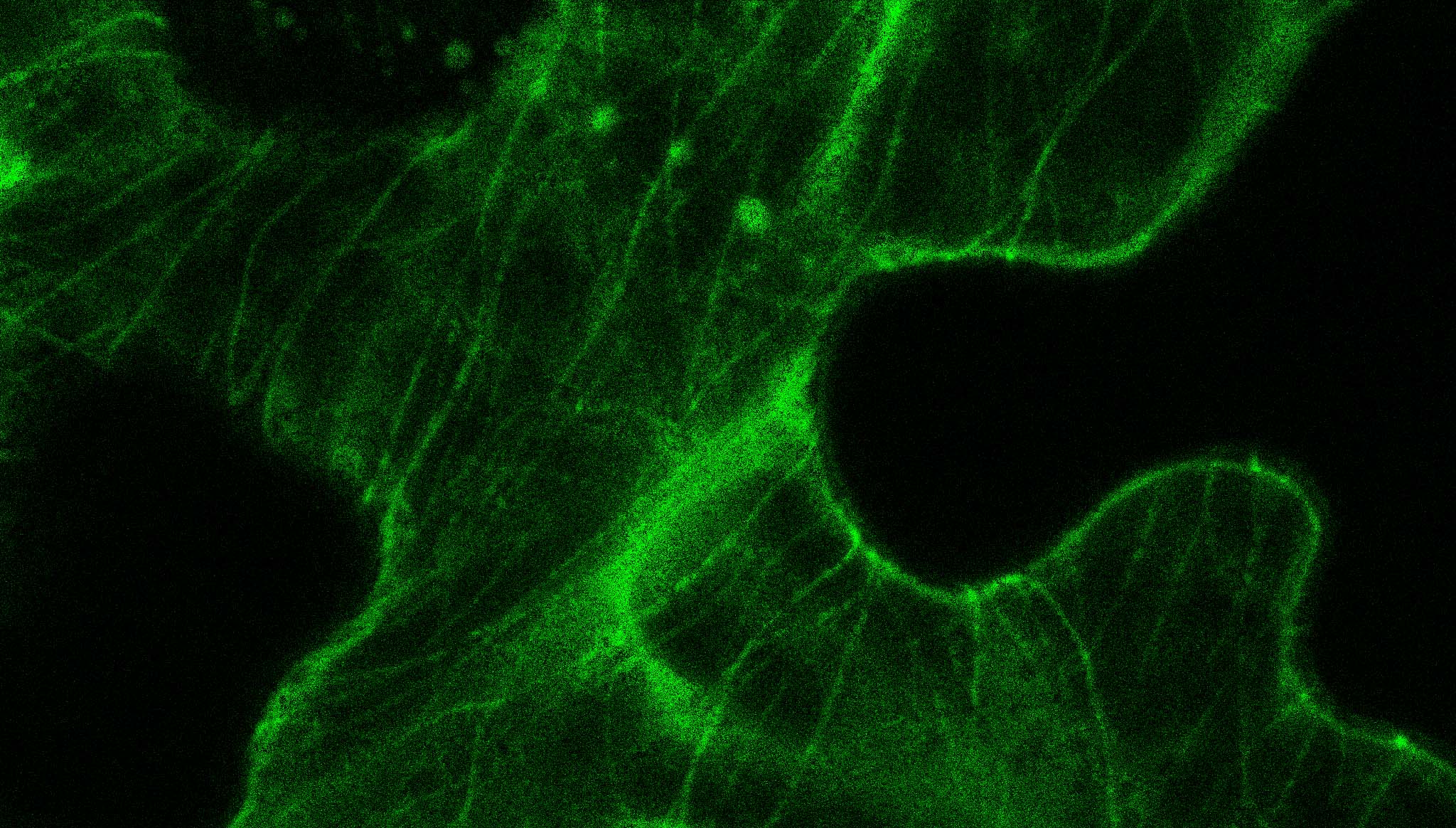

Supplement: Supplemental Information 3 [file peerj-12-18118-s003.zip › The original images for Fig.3(JPEG格式)/Fig.3a/GFP-α tubulin.jpg]

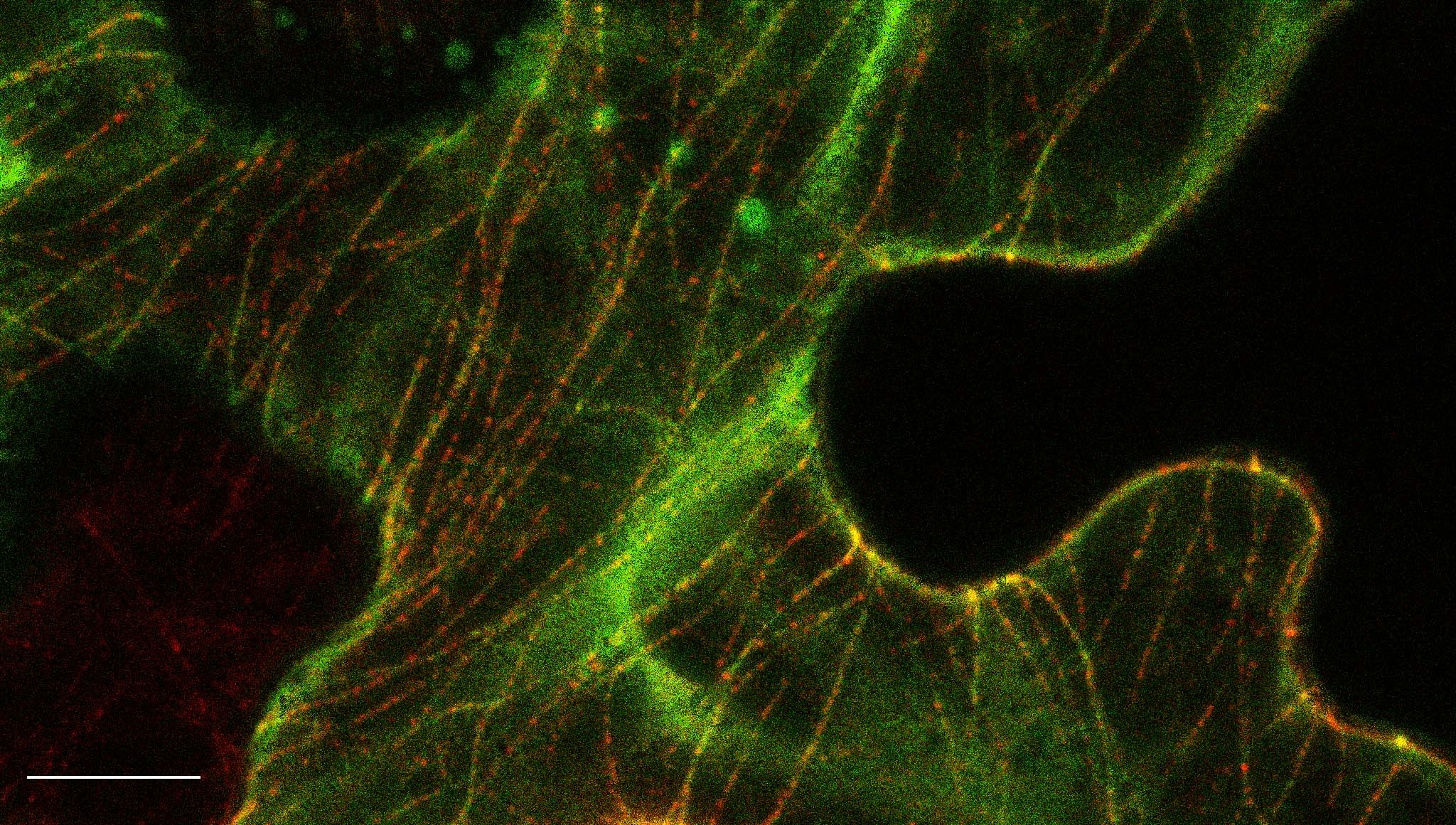

Supplement: Supplemental Information 3 [file peerj-12-18118-s003.zip › The original images for Fig.3(JPEG格式)/Fig.3a/Merge.jpg]

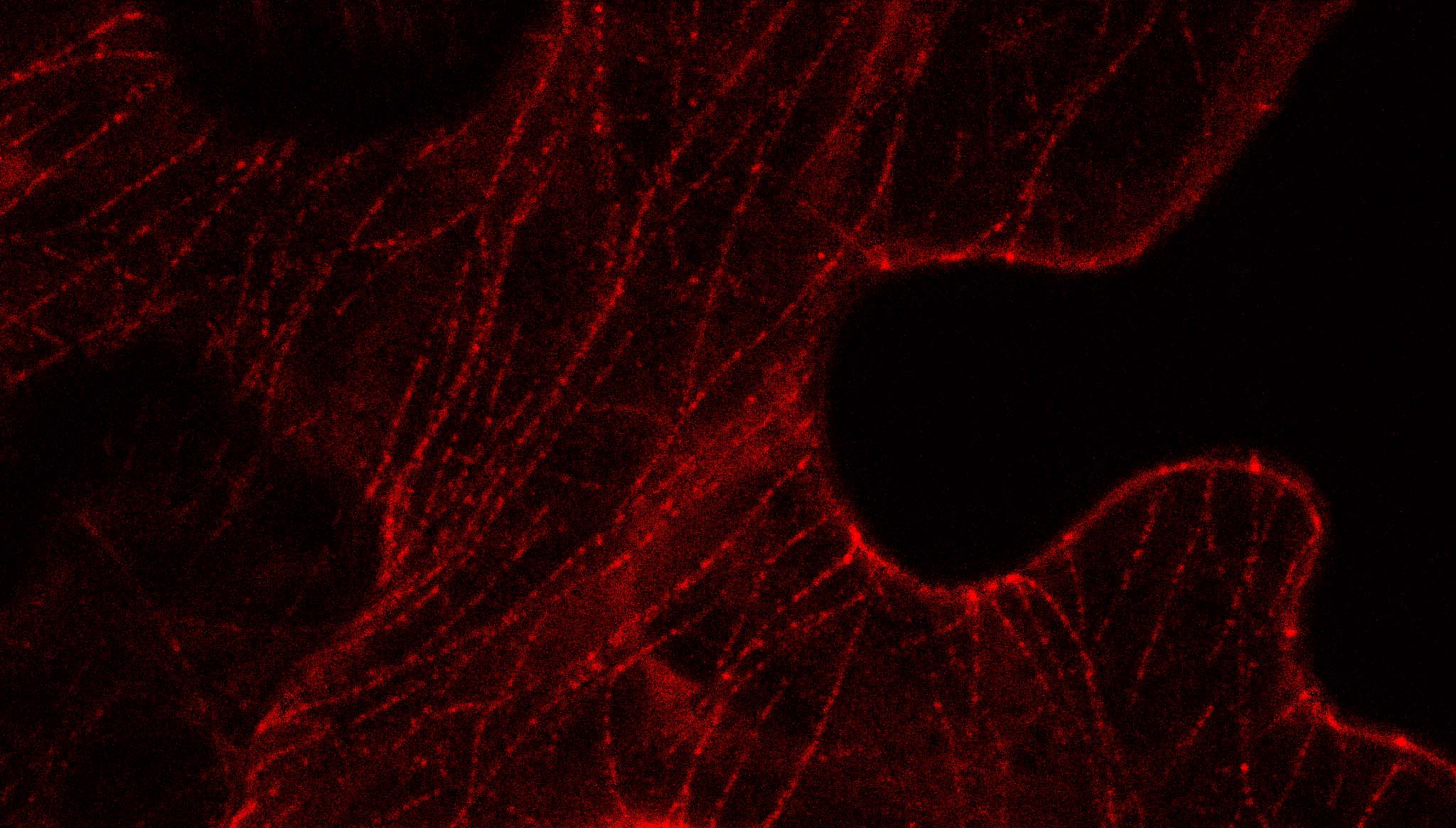

Supplement: Supplemental Information 3 [file peerj-12-18118-s003.zip › The original images for Fig.3(JPEG格式)/Fig.3a/mCherry-GL7.jpg]

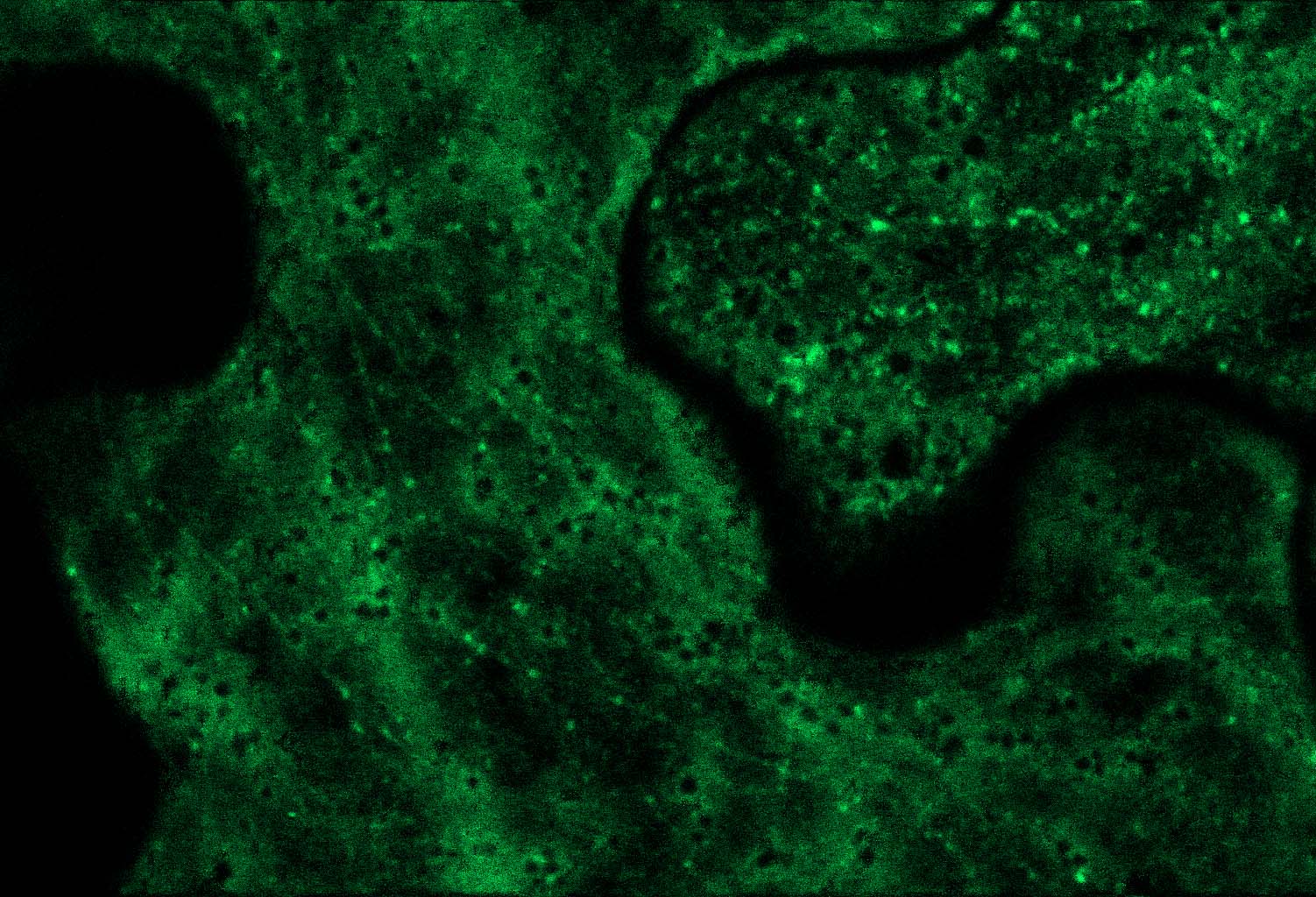

Supplement: Supplemental Information 3 [file peerj-12-18118-s003.zip › The original images for Fig.3(JPEG格式)/Fig.3b/GFP-α tubulin.jpg]

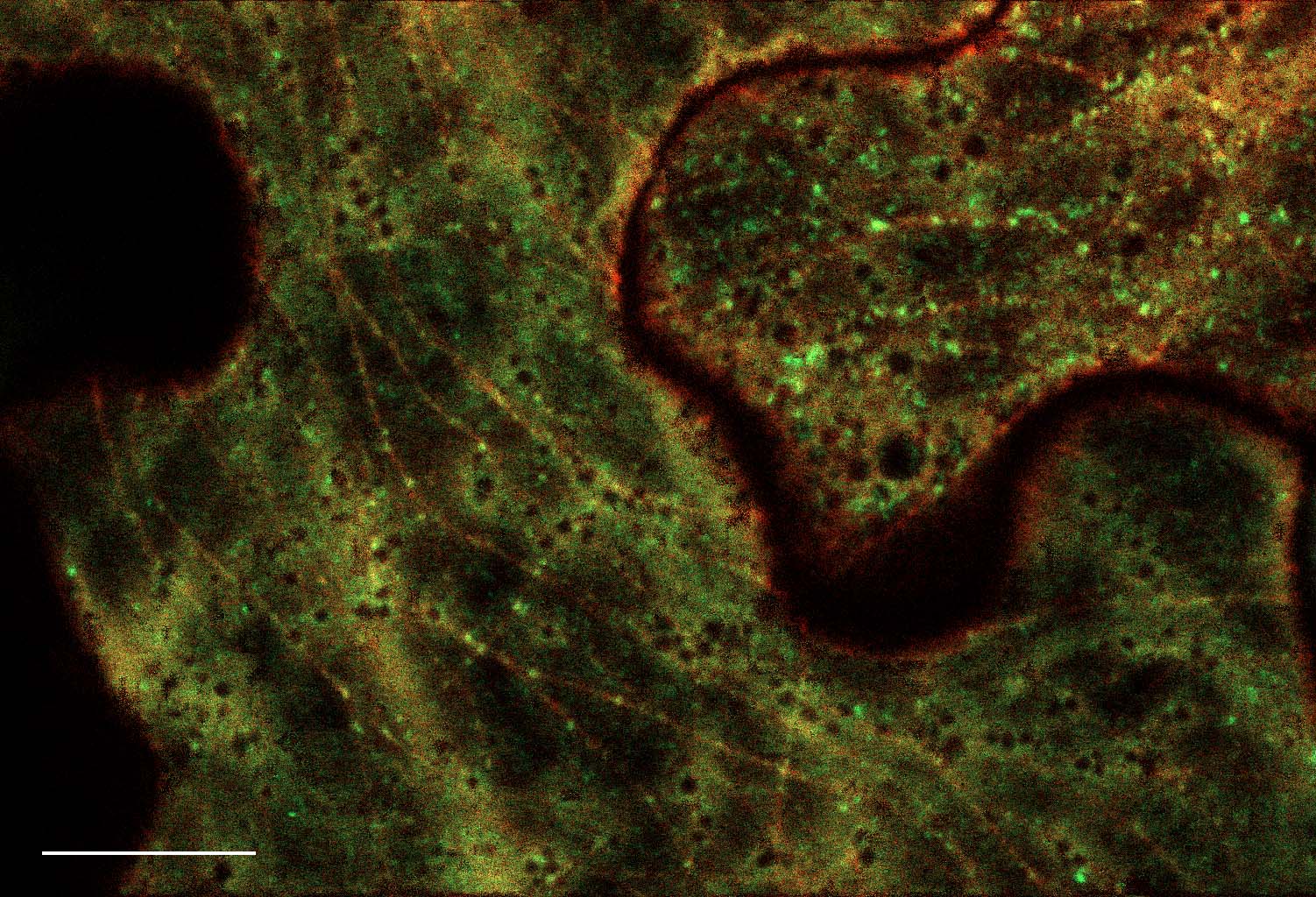

Supplement: Supplemental Information 3 [file peerj-12-18118-s003.zip › The original images for Fig.3(JPEG格式)/Fig.3b/Merge.jpg]

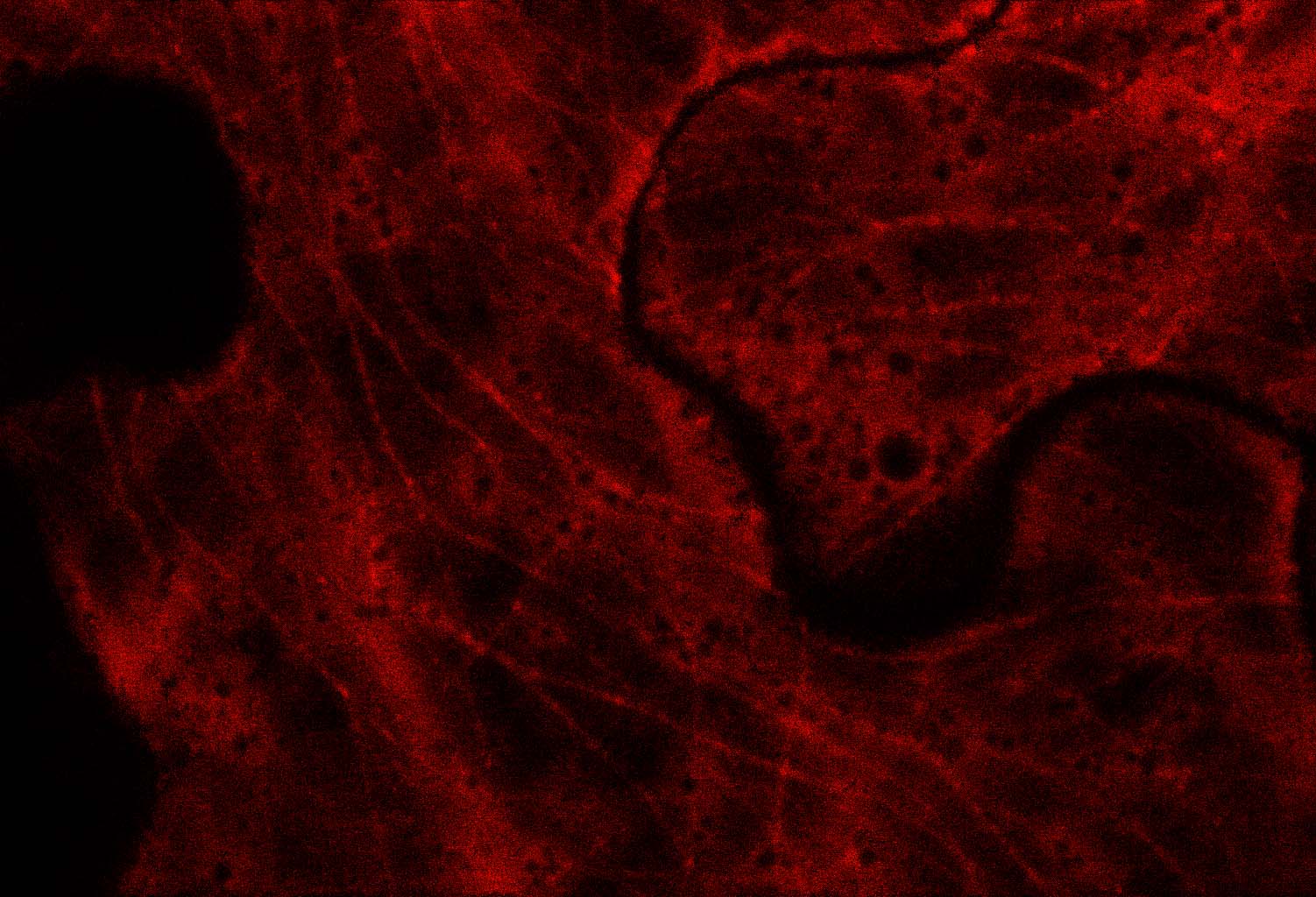

Supplement: Supplemental Information 3 [file peerj-12-18118-s003.zip › The original images for Fig.3(JPEG格式)/Fig.3b/mCherry-OsKCBP.jpg]

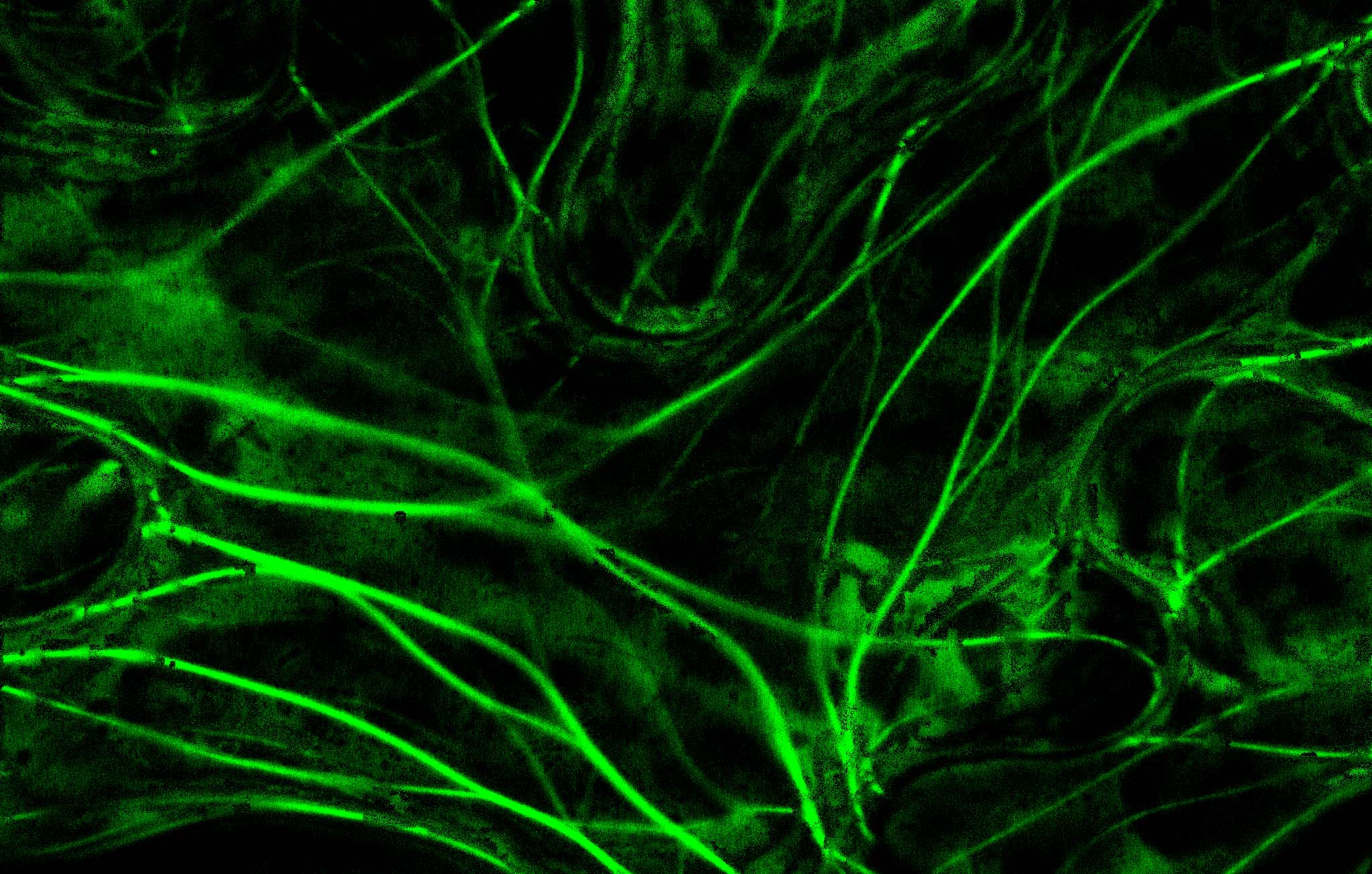

Supplement: Supplemental Information 3 [file peerj-12-18118-s003.zip › The original images for Fig.3(JPEG格式)/Fig.3c/GFP-α tubulin.jpg]

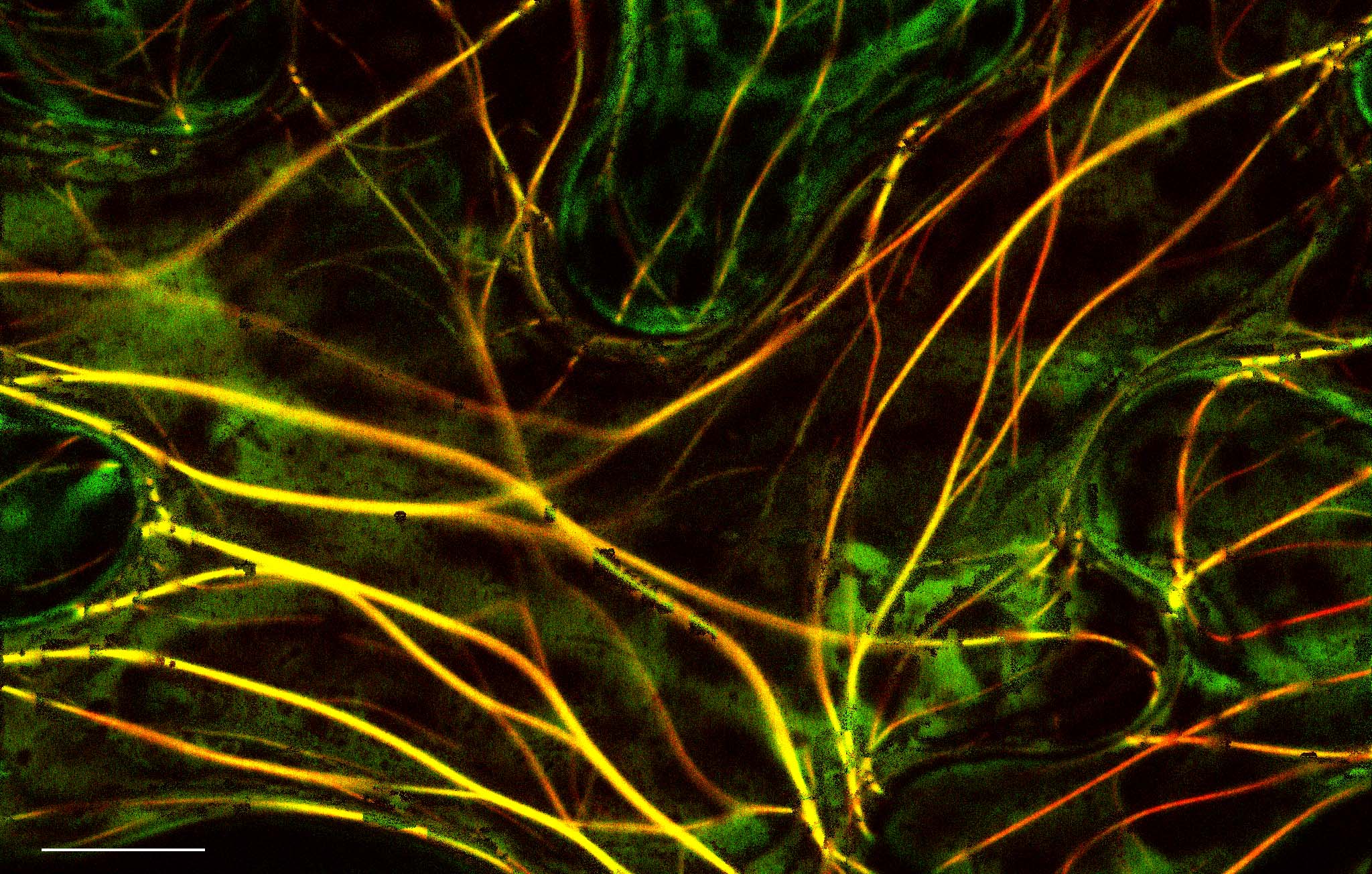

Supplement: Supplemental Information 3 [file peerj-12-18118-s003.zip › The original images for Fig.3(JPEG格式)/Fig.3c/Merge.jpg]

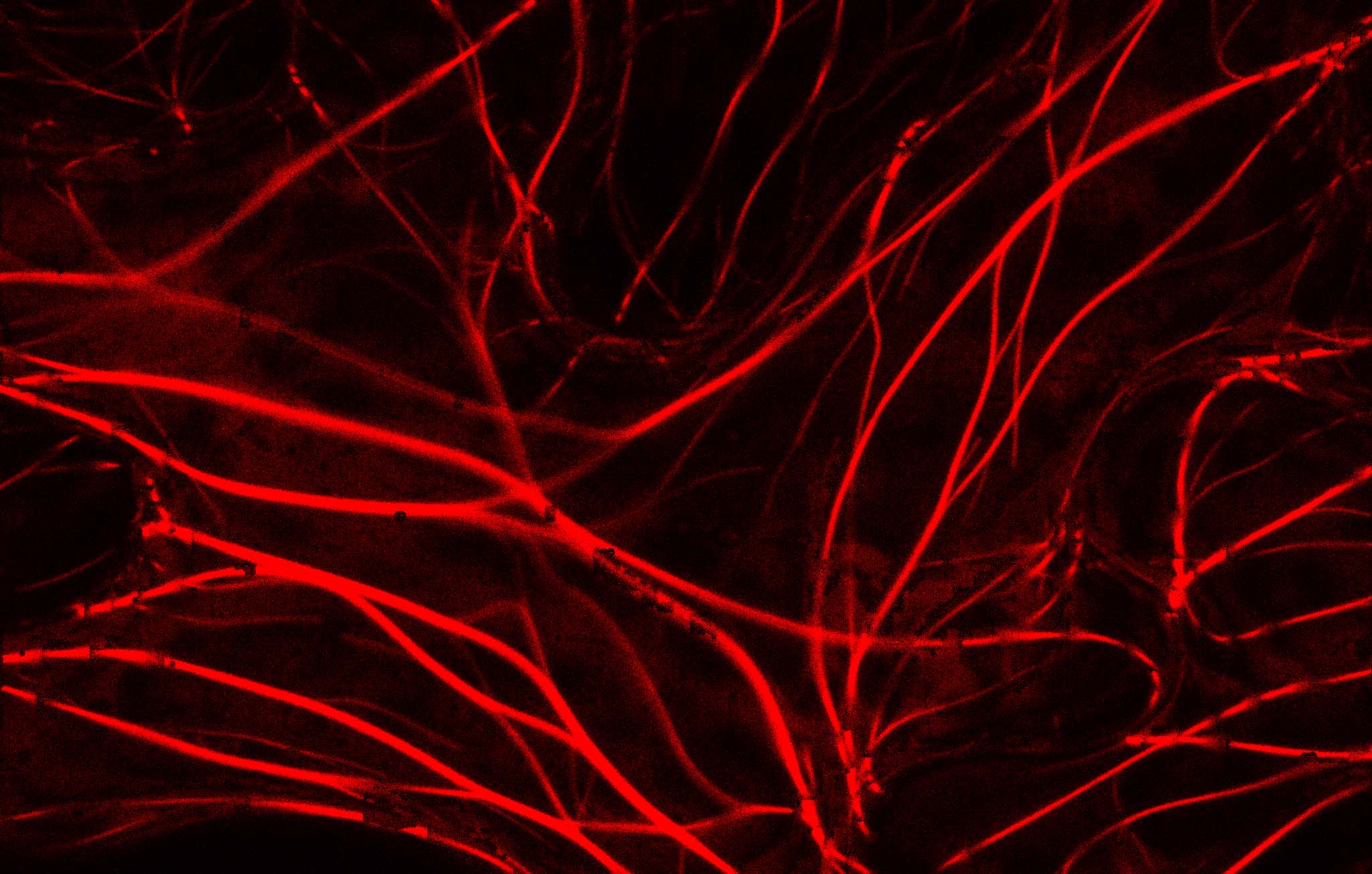

Supplement: Supplemental Information 3 [file peerj-12-18118-s003.zip › The original images for Fig.3(JPEG格式)/Fig.3c/mCherry-OsCLASP.jpg]

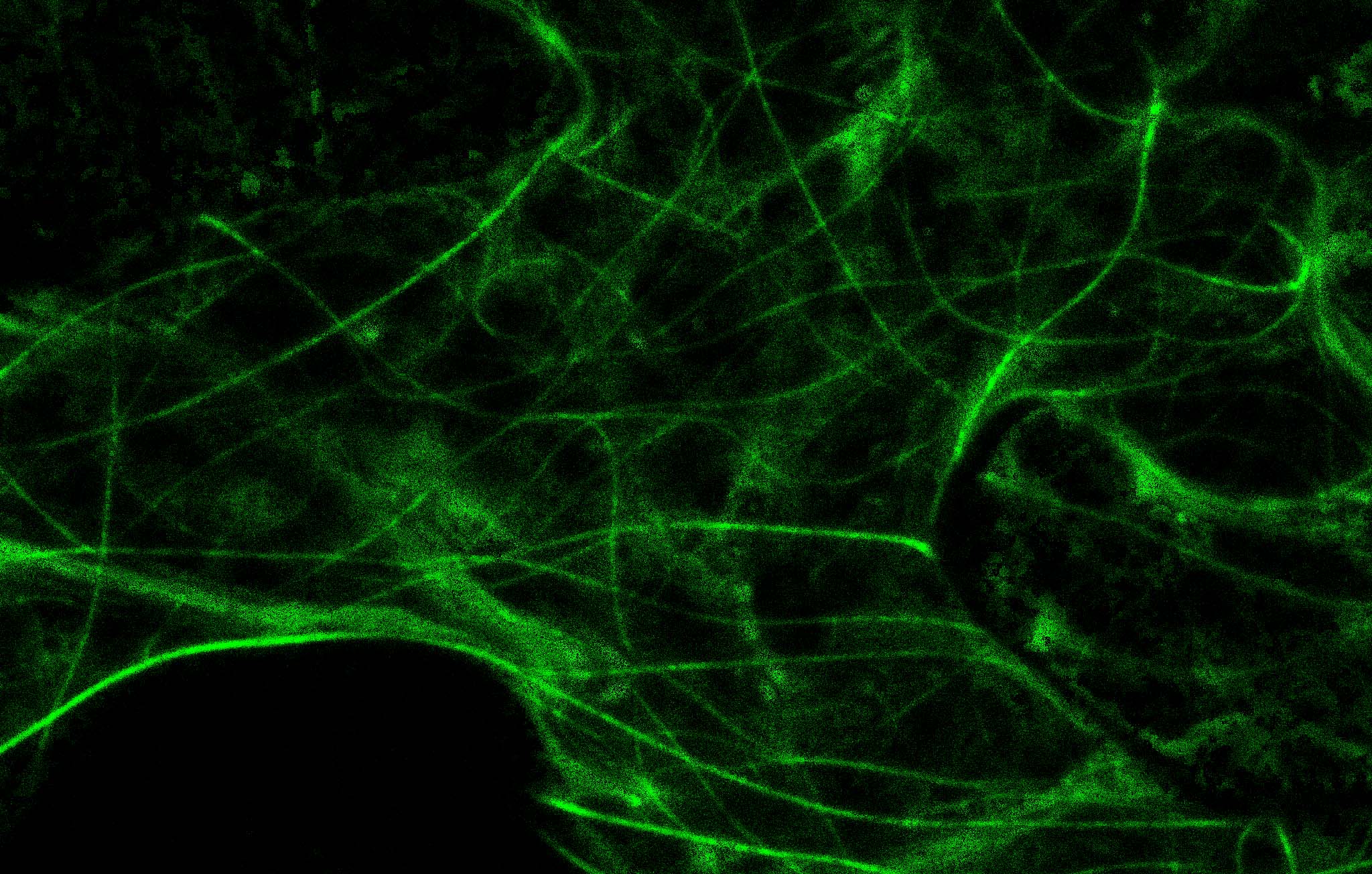

Supplement: Supplemental Information 3 [file peerj-12-18118-s003.zip › The original images for Fig.3(JPEG格式)/Fig.3d/GFP-α tubulin.jpg]

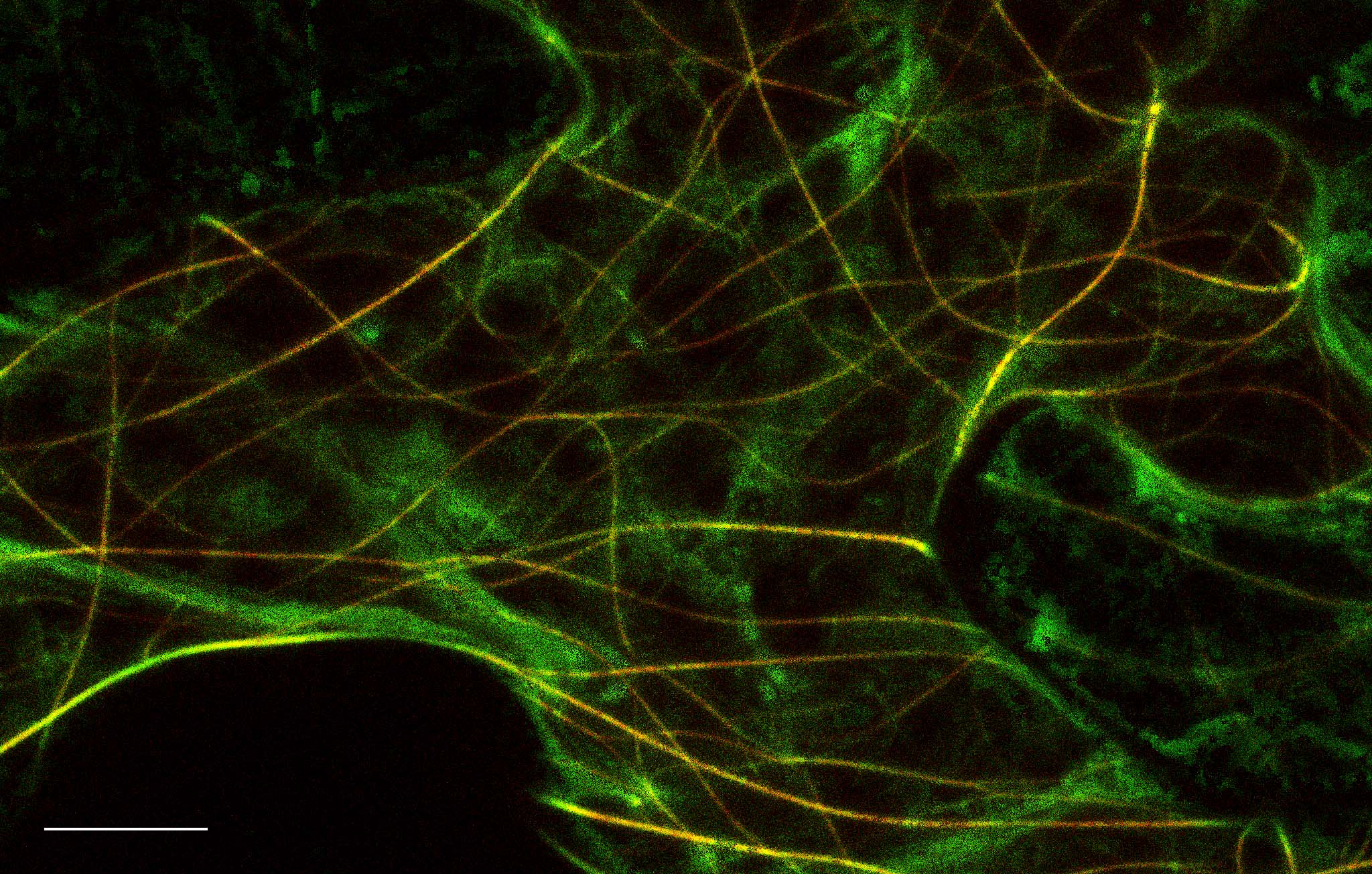

Supplement: Supplemental Information 3 [file peerj-12-18118-s003.zip › The original images for Fig.3(JPEG格式)/Fig.3d/Merge.jpg]

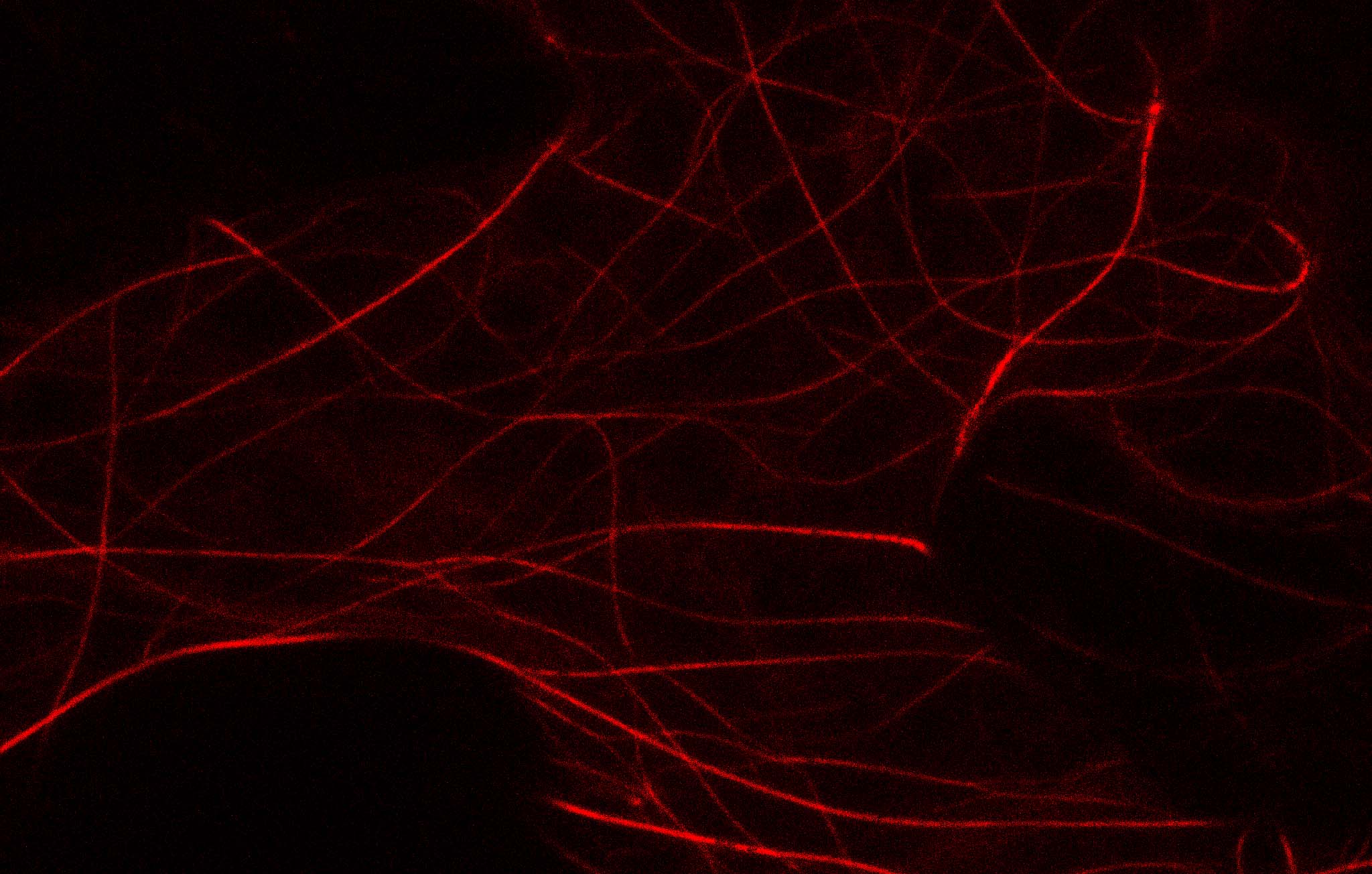

Supplement: Supplemental Information 3 [file peerj-12-18118-s003.zip › The original images for Fig.3(JPEG格式)/Fig.3d/OsMOR1-mCherry.jpg]

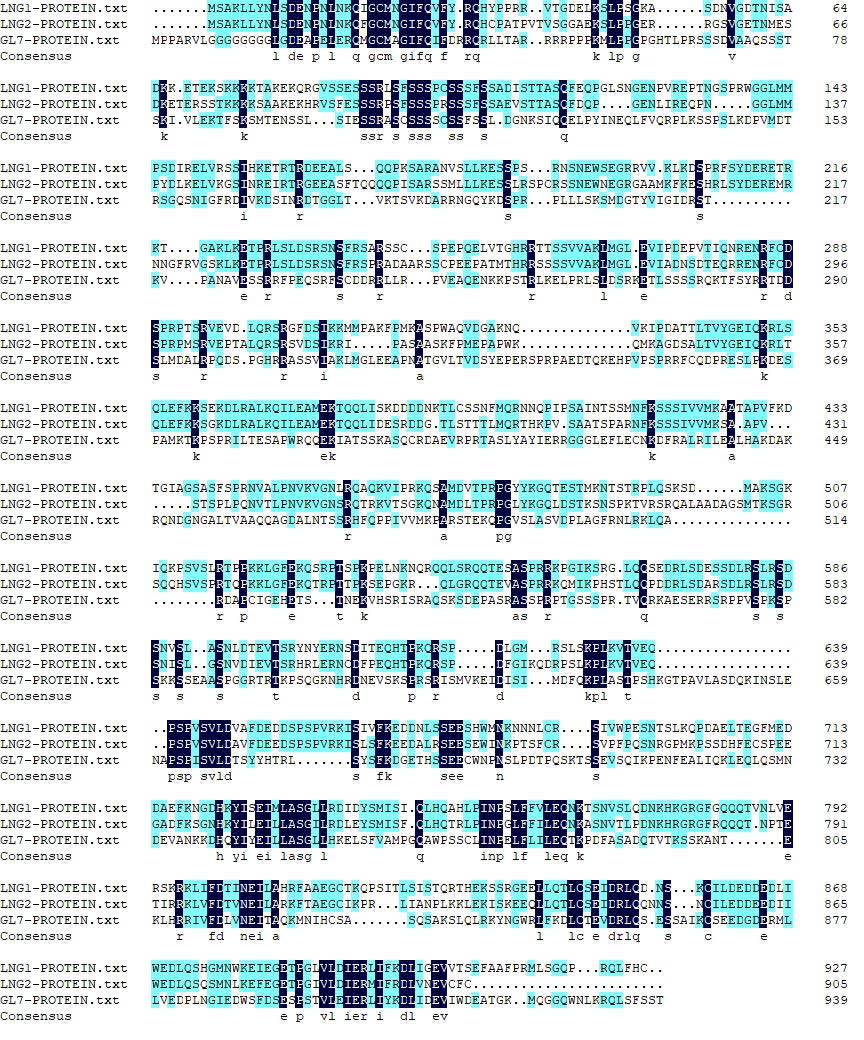

Supplement: Supplemental Information 11 — The numbers on the right indicate the position of the residues in the proteins. Identical residues were highlighted by dark blue boxes, and conserved residues by cyan boxes. DNAMAN software was used for sequence alignment (the same as below). [file peerj-12-18118-s011.jpg]

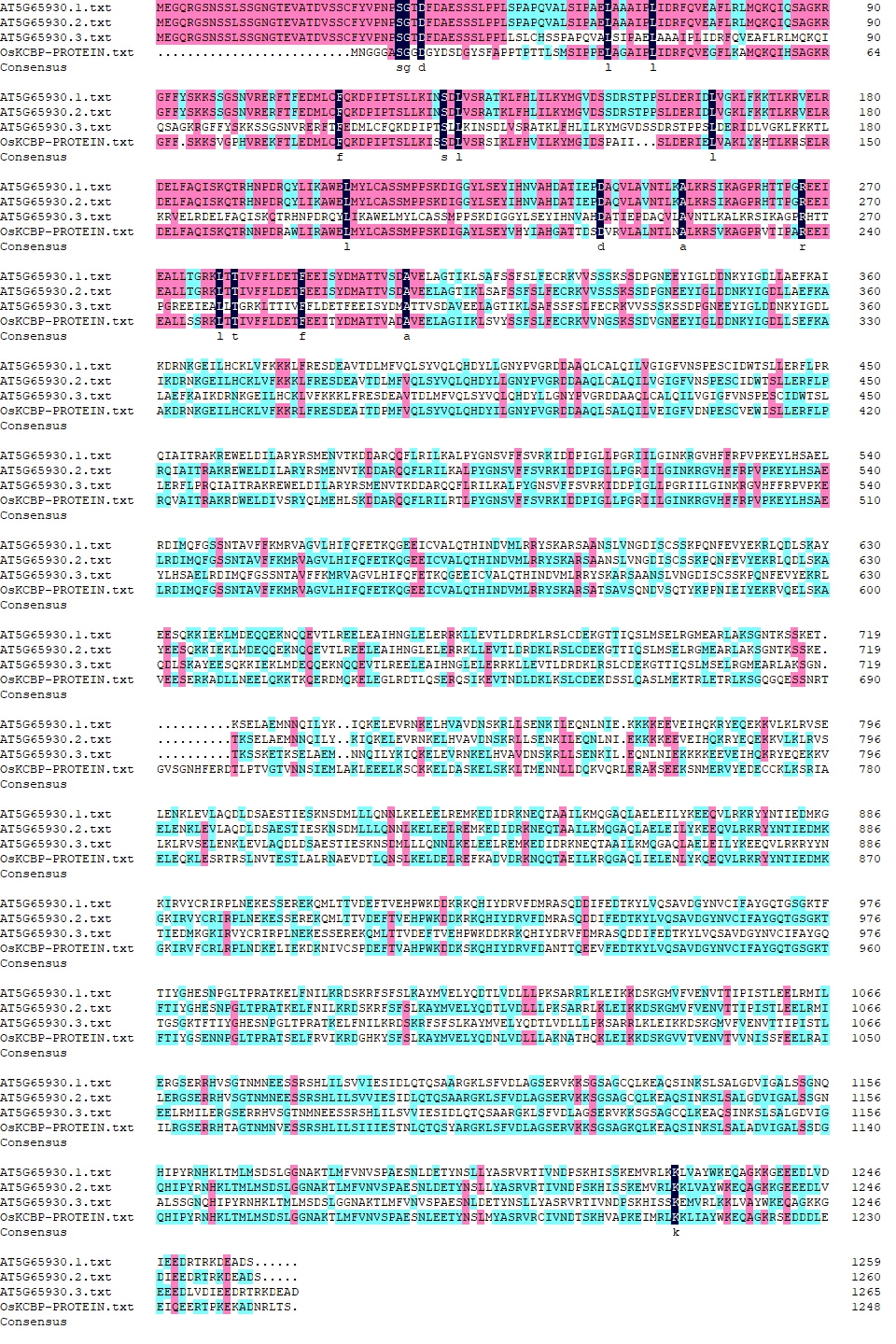

Supplement: Supplemental Information 12 — The numbers on the right indicate the position of the residues in the proteins. Identical residues were highlighted by dark blue boxes, and conserved residues by magenta and cyan boxes. [file peerj-12-18118-s012.jpg]

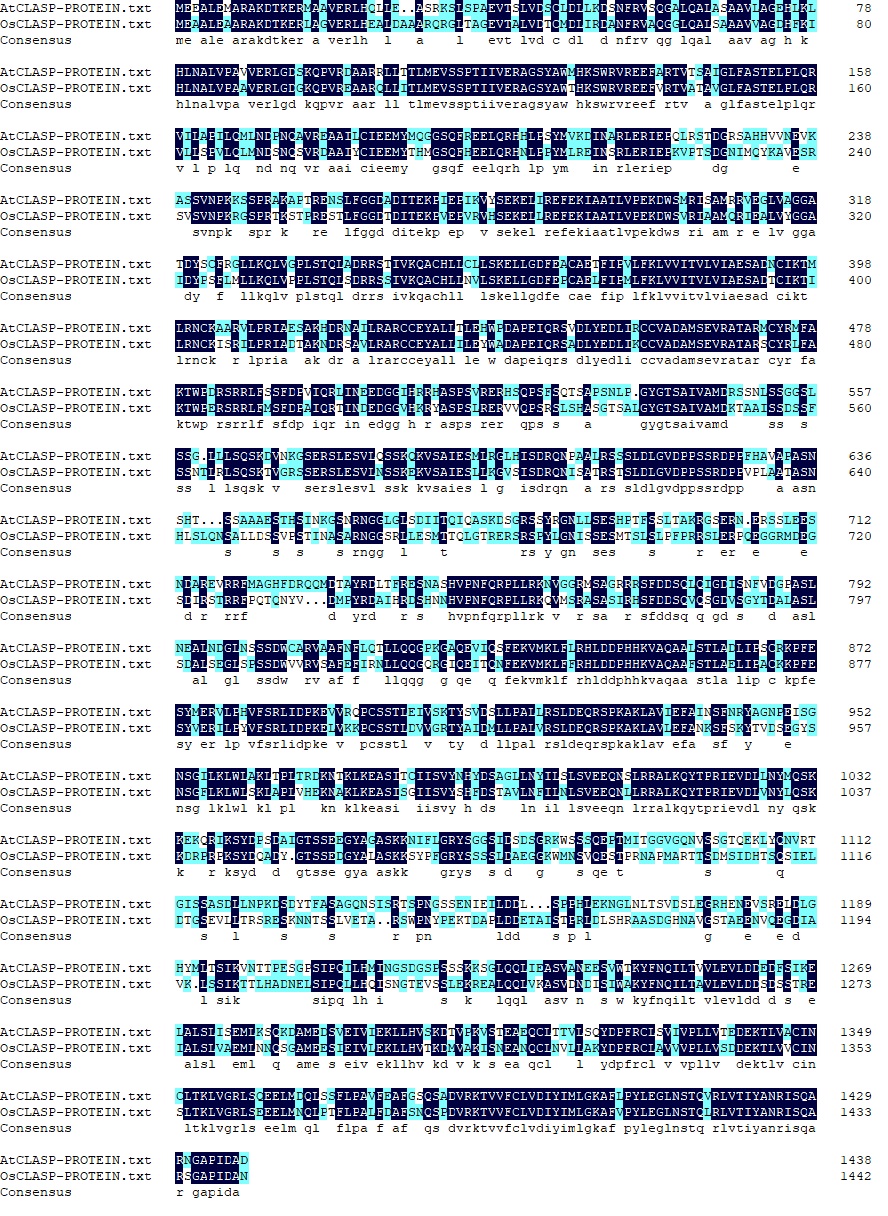

Supplement: Supplemental Information 13 — The numbers on the right indicate the position of the residues in the proteins. Identical residues were highlighted by dark blue boxes, and conserved residues by cyan boxes. [file peerj-12-18118-s013.jpg]

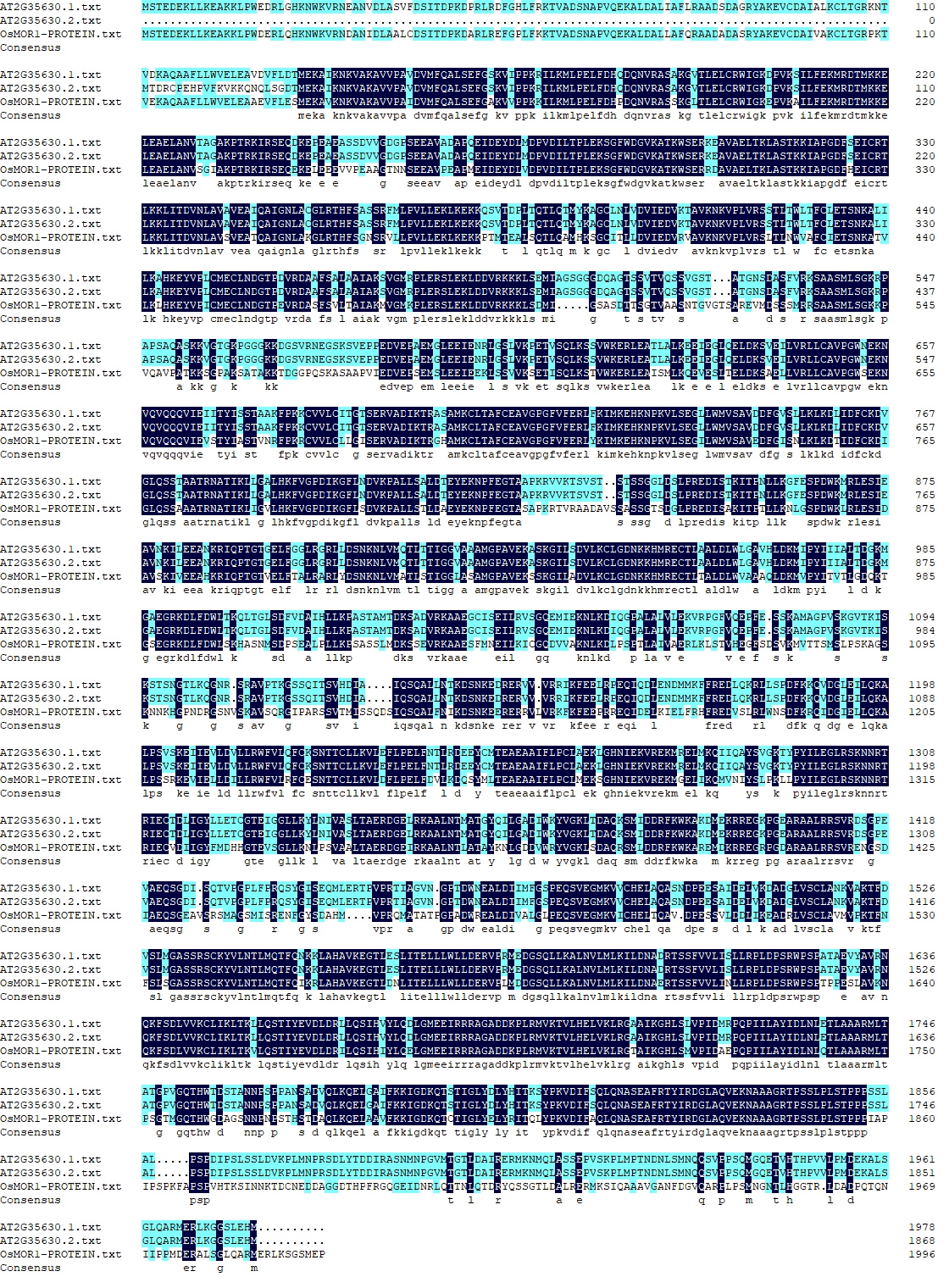

Supplement: Supplemental Information 14 — The numbers on the right indicate the position of the residues in the proteins. Identical residues were highlighted by dark blue boxes, and conserved residues by cyan boxes. [file peerj-12-18118-s014.jpg]
