# Supplementary material for: Construction and validation of co-expression vector for rice alpha tubulin and microtubule associated protein respectively fused with fluorescent proteins
Source: PeerJ. 2024 Sep 26;12:e18118. doi: 10.7717/peerj.18118 (PMC11439384; doi:10.7717/peerj.18118)
Supplement: Supplemental Information 10 [file peerj-12-18118-s010.docx]

**Table S1. Primers used in this study.**

| **Gene** | **Description** | **Primer** | **Primer specific sequence (5' - 3')** | | **Amplicon** |
| --- | --- | --- | --- | --- | --- |
| *LOC_Os07g38730* | *α tubulin* ORF | ATUB | F: | ATGAGAGAGATCATCAGCATCCACA | 1353 |
|  |  |  | R: | CTAATAGTCTTCTCCATCGTCGTT |  |
| *eGFP* | *eGFP* sequence | eGFP | F: | ATGGTGAGCAAGGGCGA | 717 |
|  |  |  | R: | CTTGTACAGCTCGTCCA |  |
| *mCherry* | *mCherry* sequence | MCH | F: | ATGGTGAGCAAGGGCGAGGAG | 711 |
|  |  |  | R: | TTACTTGTACAGCTCGTCCATGCCG |  |
| *NOS* | *NOS* terminator from pCambia1391 | NOS1 | F: | ATTGGTGACCAGCTCGAATTT | 299 |
|  |  |  | R: | AACACTGATAGTTTAATTCCCGATCTAGT |  |
| *NOS* | *NOS* terminator from pBI221-H2B-mCherry | NOS2 | F: | GAATTTCCCCGATCGTCAAACATTTG | 262 |
|  |  |  | R: | GATCTAGTAACATAGATGACACCGC |  |
| *35S* | *35S* promoter sequence | 35S | F: | GGTCCCCAGATTAGCCTTTTCAAT | 835 |
|  |  |  | R: | TCTCCAAATGAAATGAACTTCCTT |  |
| *LOC_Os07g41200* | *GL7* ORF | GL7 | F: | ATGCCTCCGGCGAGGGTG | 2823 |
|  |  |  | R: | TCAGCTTGTACTACTAAATGACAGCTGC |  |
| *LOC_Os04g57140* | *OsKCBP* ORF | KCBP | F: | ATGAACGGTGGCGGCGC | 3747 |
|  |  |  | R: | TCAGCTAGTCAAGCGATTATCTGCTTT |  |
| *LOC_Os04g42840* | *OsCLASP* ORF | CLASP | F: | ATGGAGGCGGCGCTGGAGG | 4332 |
|  |  |  | R: | TCATTGGTTAGCATCAATCGGAGCG |  |
| *LOC_Os01g60040* | *OsMOR1* ORF | MOR1 | F: | ATGTCGACGGAGGACGAGAAGC | 5991 |
|  |  |  | R: | AAGTGGTTCCATAGACCCACTC |  |
